# EngAGE via Alexa for Older Adults and Caregivers: Design, Utilization, and Impact of Socially Motivated Exercise

**DOI:** 10.1093/geroni/igaa057.2219

**Published:** 2020-12-16

**Authors:** Megan Huisingh-Scheetz, Roscoe Nicholson, Chelsea Smith, Saira Shervani, Yadira Montoya, Louise Hawkley

**Affiliations:** 1 University of Chicago, Riverside, Illinois, United States; 2 University of Chicago, Chicago, Illinois, United States; 3 NORC at the University of Chicago, Chicago, Illinois, United States

## Abstract

EngAGE is a technology-based program leveraging Alexa that encourages older adult (OA) activity and socialization from home while empowering caregivers to support them. EngAGE delivers daily, in-home, NIA Go4Life exercise routines with instructions, pictures and music via Alexa Echo Shows or Fire Tablets to OAs. Caregivers use EngAGE to view scheduled exercises, follow progress, and send encouraging messages that are read aloud to OAs by Alexa. We will discuss the strategic co-design of EngAGE with OAs and caregivers and the utilization and functional impact of EngAGE over a 12-week feasibility and usability study (n=10 OA + caregiver pairs). Preliminary analyses revealed improvement in upper (mean grip strength change = +1.3 kg, paired t-test p=0.34) and lower (5-repeated chair stand time change = -2.3 seconds, paired t-test p=0.02) body strength. Discussion of focus group data will cover themes of perceived benefits, user experience, drivers/barriers to usage and desired features for EngAGE.

